# The correlation between the atherogenic index of plasma and aortic valve calcification in the general population: a retrospective observational cohort study

**DOI:** 10.3389/fendo.2026.1846100

**Published:** 2026-06-04

**Authors:** Bolun Jiao, Rui Yu, Lei Li, Yangmin Hao, Guoli Du, Sheng Jiang

**Affiliations:** 1 The First Clinical Medical College, Xinjiang Medical University, Urumqi, Xinjiang, China; 2 Health Management Center, The First Affiliated Hospital of Xinjiang Medical University, Urumqi, Xinjiang, China; 3 Department of Endocrinology and Metabolism, The First Affiliated Hospital of Xinjiang Medical University, Urumqi, Xinjiang, China

**Keywords:** aortic valve calcification, atherogenic index of plasma, general population, receiver operating characteristic curve, sensitivity analysis

## Abstract

**Objective:**

This study aims to explore the correlation between atherogenic index of plasma (AIP) and the risk of aortic valve calcification (AVC).

**Method:**

This single-center retrospective cohort study included 1,752 participants from the First Affiliated Hospital of Xinjiang Medical University, from May 27, 2023, to Ju\ly 27, 2025. Participants were divided into two groups based on AVC at follow-up: non–AVC group (n = 1,463) and AVC group (n = 289). Multivariate logistic regression, subgroup and sensitivity analyses, and receiver operating characteristic (ROC) curve analysis evaluated the evaluate the correlation between AIP and AVC.

**Results:**

Multivariable logistic regression analysis showed that each 1-unit increase in AIP was associated with a 146.0% higher risk of AVC (OR = 2.460, 95% CI 1.525–3.969). When participants were grouped by AIP quartiles, compared with the lowest quartile (Q1), the risk of AVC in the Q2, Q3, and Q4 groups was 1.623-fold (95% CI 1.080–2.439, P = 0.020), 2.405-fold (95% CI 1.622–3.567, P < 0.001), and 2.319-fold (95% CI 1.564–3.439, P < 0.001), respectively. Subgroup analysis indicated that higher AIP levels remained significantly associated with an increased risk of AVC across most subgroups (all P < 0.05). Sensitivity analysis, after excluding participants with eGFR < 60 mL/min/1.73 m², showed that each 1-unit increase in AIP was associated with a 143.2% higher risk of AVC. In that analysis, the AVC risk in Q2, Q3, and Q4 remained 2.086-fold, 2.928-fold, and 3.172-fold that of Q1, respectively. When grouped by the median AIP, the high-AIP group had a 1.996-fold higher risk; when grouped by the mean AIP, the risk was 2.067-fold higher. Using AIP tertiles, the Q2 and Q3 groups had 2.151-fold and 2.807-fold higher risks, respectively. ROC curve analysis indicated that AIP had moderate diagnostic value for AVC risk (AUC = 0.760, 95% CI 0.732–0.789). In contrast, the AUCs for triglycerides (0.594) and total cholesterol (0.538) were significantly lower than that of AIP (DeLong test, both P < 0.05).

**Conclusion:**

Higher levels of AIP are significantly associated with an increased risk of AVC, indicating that AIP holds important clinical value as an associative biomarker for AVC.

## 1 Introduction

AVC is a common progressive disease that poses a heavy burden on global health, closely linked to an increased incidence of cardiovascular events and higher mortality rates (1, 2). The prevalence of the disease sharply rises with age, with an estimated 25% of individuals over 65 affected and as many as 50% of those over 85 impacted (3, 4). Currently, known risk factors for AVC include old age, male gender, hypertension, dyslipidemia, smoking, lipoprotein(a), and sex-specific factors; however, some potential indicators and risk factors still require further analysis and exploration (5–7).

Existing evidence indicates that AIP is a reliable and independent biomarker for cardiovascular diseases (8). AIP comprehensively reflects the metabolic status of triglycerides (TG) and high-density lipoprotein cholesterol (HDL-C) and can indicate the degree of lipoprotein dysregulation as well as the burden of atherosclerosis (9). Elevated AIP levels are closely associated with an increased risk of major adverse cardiovascular events (MACEs), such as coronary heart disease, myocardial infarction, and stroke, and provide additional predictive value beyond traditional lipid indicators (10). A meta-analysis by Rabiee et al. indicates that the risk of MACEs in the highest AIP group is 63% higher than in the lowest group (11). A machine learning cohort study confirmed that AIP is an independent predictor of prognosis in acute coronary syndrome (ACS) patients (12). Furthermore, a growing body of evidence has established AIP as an independent predictor of vascular calcification, including coronary artery calcification (CAC) and abdominal aortic calcification (AAC). Li et al.’s study showed that AIP levels are significantly positively correlated with the risk of CAC (13); moreover, a cross-sectional study found a correlation between AIP and AAC in the study population. For each unit increase in the AIP index, the likelihood of AAC doubles (14). AIP is characterized by the accumulation of triglyceride-rich lipoproteins and a reduction in HDL-C. It may drive the differentiation of vascular smooth muscle cells into an osteoblast-like phenotype through exacerbating oxidative stress and promoting inflammatory microenvironments (9, 15, 16). Dysregulated lipid metabolism not only shapes a microenvironment conducive to calcification but also directly participates in the mineralization process through functional changes in apolipoprotein A-I, a key component of high-density lipoprotein (17–19). In addition, AIP may also regulate the activity of lipoprotein-associated enzymes (such as autocrine motility factors), further participating in the regulation of the calcification signaling pathway (20).

Although AIP has been extensively documented as an independent correlate of vascular calcification, including CAC and AAC, it is crucial to recognize that AVC is pathologically distinct from arterial calcification. Unlike intimal atherosclerosis in coronary or aortic diseases, AVC primarily involves the fibrosa layer of the valve cusp, driven by a unique combination of mechanical stress, chronic inflammation, and osteogenic differentiation of valvular interstitial cells (21). The hemodynamic environment of the aortic valve — characterized by cyclic stretch, shear stress, and pressure gradients — differs fundamentally from that of the arterial wall (22). Therefore, findings from vascular calcification studies cannot be directly extrapolated to AVC. Despite the well-established role of AIP in vascular calcification, direct evidence linking AIP to AVC in the general population remains scarce. Only one recent study has explored this relationship, but it was limited to elderly patients with degenerative valvular heart disease, with a small sample size and restricted generalizability (23). More importantly, no study has systematically addressed whether AIP provides incremental clinical value beyond traditional lipid parameters or existing vascular calcification biomarkers for identifying individuals at higher risk of AVC. Given that AVC shares some but not all risk factors with vascular calcification, there is an unmet need to determine whether AIP — as a composite lipid index — offers unique, non-redundant information for AVC risk assessment.

Therefore, based on the above research background, to further enrich this field of study, this research aims to evaluate the correlation between AIP and AVC in the general population. It seeks to provide new clues and theoretical basis for risk assessment and clinical management of AVC.

## 2 Research methods

### 2.1 Study population

In this single-center retrospective cohort study, participants were included from the First Affiliated Hospital of Xinjiang Medical University from May 27, 2023, to July 27, 2025, based on the inclusion and exclusion criteria listed below. Inclusion criteria included: (1) The study period was from May 27, 2023, to July 27, 2025, with at least one visit record at the First Affiliated Hospital of Xinjiang Medical University and complete inpatient or outpatient electronic medical record data; (2) Age ≥ 18 years, with complete age records at the time of enrollment; (3) Availability of lipid profile test results (at minimum including triglycerides and high-density lipoprotein cholesterol). Exclusion criteria included: (1) Severe liver dysfunction meeting any of the following conditions (Laboratory tests showing alanine aminotransferase (ALT) or aspartate aminotransferase (AST) > 5 times the upper limit of normal (ULN) and total bilirubin (TBil) > 3 times (ULN); (2) Severe renal dysfunction, with an estimated glomerular filtration rate (eGFR) < 30 mL/min/1.73 m², or long-term maintenance on hemodialysis or peritoneal dialysis; (3) Presence of severe valvular heart disease or any history of aortic valve and/or other heart valve replacement surgery (including mechanical valves, bioprosthetic valves, transcatheter aortic valve replacement [TAVR], or surgical valvuloplasty); (3) Any organ system active malignant tumors diagnosed by pathology or typical imaging (including but not limited to solid tumors and hematologic tumors, etc.); (4) Congenital heart structural abnormalities clearly diagnosed by cardiac ultrasound or previous surgical records, including but not limited to: bicuspid aortic valve, unicuspid aortic valve, quadricuspid aortic valve, subaortic stenosis, ventricular septal defect, atrial septal defect, Tetralogy of Fallot, etc.; (5) Presence of acute infectious diseases, including but not limited to: pneumonia (diagnosed by imaging), sepsis (positive blood culture or clinical diagnostic criteria), acute urinary tract infection with fever (temperature ≥ 38.5 °C), acute biliary tract infection, acute pancreatitis, appendicitis, cellulitis, etc., requiring hospitalization or intravenous antimicrobial treatment, or laboratory tests indicating acute inflammatory state: high-sensitivity C-reactive protein (hs-CRP) > 10 mg/L with clinical symptoms, such as fever, elevated white blood cell count (WBC) > 10.0 × 10^9^/L, or increased neutrophil percentage > 75%; (6) Thyroid dysfunction (including hyperthyroidism or hypothyroidism); (7) Pregnant or lactating women; (8) Familial hypercholesterolemia; (9) Severe hematologic disorders (such as leukemia or lymphoma). After applying the inclusion and exclusion criteria, a total of 1,752 participants were consecutively included in this study for further analysis. The study was reviewed and approved by the Ethics Committee for Human Research at the First Affiliated Hospital of Xinjiang Medical University (Approval No.: 2506-34) and complied with the relevant requirements of the World Medical Association’s Declaration of Helsinki. Due to the retrospective nature of the study and the use of de-identified data, the requirement for written informed consent was waived by the ethics committee.

### 2.2 Collection and definition of variables

In this study, demographic data, anthropometric measurements, comorbidity information, and blood biomarker data were collected from the electronic medical records system of the First Affiliated Hospital of Xinjiang Medical University. Demographic data included age, sex, and smoking status. Smoking was defined as current or past smoking, regardless of whether cessation had occurred. Comorbidity data included hypertension, diabetes, dyslipidemia, obesity, and hyperuricemia. According to the European Society of Cardiology (ESC) (24), hypertension is defined as: systolic blood pressure (SBP) ≥ 140 mmHg or diastolic blood pressure (DBP) ≥ 90 mmHg, or a documented history of hypertension with current antihypertensive treatment. According to the American Diabetes Association diagnostic criteria for diabetes (25), diabetes was defined as: glycated hemoglobin (HbA1c) ≥ 6.5%, fasting plasma glucose (FPG) ≥ 7.0 mmol/L, 2-hour postprandial glucose (2hPPG) ≥ 11.1 mmol/L, or a documented history of diabetes with current antidiabetic treatment. According to the Chinese Guidelines for the Prevention and Treatment of Dyslipidemia in Adults (26), dyslipidemia is defined as: fasting TG ≥ 2.3 mmol/L, low-density lipoprotein cholesterol (LDL-C) ≥ 4.1 mmol/L, total cholesterol (TC) ≥ 6.2 mmol/L, or HDL-C < 1.0 mmol/L. According to ESC Consensus Statement on Obesity and Chronic Kidney Disease diagnostic criteria (27), obesity is defined as: body mass index (BMI) ≥ 30 kg/m^2^. According to international expert consensus and the Chinese Guidelines for the Diagnosis and Treatment of Hyperuricemia and Gout, hyperuricemia is defined as: serum uric acid (SUA) level ≥ 420 μmol/L (28, 29).

Anthropometric measurements include height, weight, BMI, SBP, and DBP. Here, BMI = weight (kg)/height² (m²). Blood biomarker data include WBC, neutrophil count, lymphocyte count, monocyte count, platelet count, hemoglobin, hematocrit, red blood cell distribution width (RDW), ALT, AST, albumin, TBil, FPG, HbA1c, TC, TG, HDL-C, LDL-C, serum creatinine (Scr), SUA, eGFR, free triiodothyronine (FT3), free thyroxine (FT4), 1,25-dihydroxy vitamin D3 (1,25(OH)_2_D_3_), and homocysteine. All these blood indicators were obtained by drawing antecubital venous blood from participants after an 8–12 hour fast. Blood glucose and lipid parameters were measured using the ROCHE COBAS C702 fully automated biochemical analyzer. TG and TC were measured using the cholesterol oxidase method and the immunoturbidimetric method, respectively, with units in mmol/L; FPG was measured using the hexokinase method, with units in mmol/L. During the testing process, strict quality control procedures were followed to ensure the accuracy of the results.

### 2.3 Definition and classification of AIP

In this study, AIP is defined as the logarithm of the ratio of TG to HDL-C, i.e., AIP = log_10_(TG (mmol/L)/HDL-C (mmol/L)) (30). The study was analyzed using raw continuous variables and then grouped based on several different AIP cut-off values. The groups were categorized according to the tertiles of AIP: T1 (AIP ≤ -0.06, n = 579); T2 (-0.06 < AIP ≤ 0.20, n = 591); T3 (AIP > 0.20, n = 582). Based on the median, there were two groups: Low AIP (≤ 0.07, n = 876); High (AIP > 0.07, n = 876). Based on the average value, two groups were formed: Low AIP (≤ 0.08, n = 907); High (AIP > 0.08, n = 845). The groups were also categorized by the quartiles of AIP: Q1 (AIP ≤ -0.14, n = 432); Q2 (-0.14 < AIP ≤ 0.07, n = 444); Q3 (0.07 < AIP ≤ 0.27, n = 428); Q4 (0.27 < AIP, n = 448).

### 2.4 Definition and detection methods of aortic valve calcification

AVC is usually diagnosed through echocardiography. Echocardiography shows localized or diffuse echogenic enhancement of the valve along with valve thickening (≥ 1 mm), with unrestricted leaflet motion, a valve orifice area ≥ 3 mm, and transvalvular blood flow velocity < 2.0 m/s, indicating AVC (4). The participants were divided into two groups based on AVC: the non–AVC group (n = 1,463) and the AVC group (n = 289).

### 2.5 Statistical methods

All statistical analyzes were uniformly performed using SPSS 26.0. Before formal analysis, a completeness assessment of the original data was conducted. For variables with a proportion of missing values < 30%, mean imputation was applied to fill in missing data; for variables with missing proportion ≥ 30%, the variable was excluded from further multivariate regression analyses. For continuous variables, normalcy was assessed using the Shapiro-Wilk test. Continuous variables that did not follow a normal distribution were presented as median (interquartile range). The Mann-Whitney U test was used to compare differences between two groups, while the Kruskal-Wallis test was used to compare differences between three groups. Categorical variables were presented as frequencies (percentages), and comparisons between two groups were performed using the Chi-square test or Fisher’s exact test. A univariate logistic regression analysis was used to assess the correlation between each variable and AVC, and variables with P < 0.05 were selected to construct three multivariate regression models to further validate the independent association between AIP and AVC. Among them, Model 1 only adjusted for age and sex; Model 2, based on Model 1, further adjusted for monocyte count, RDW, platelet count, albumin, TBIL, FPG, and HbA1c; Model 3, based on Model 2, further adjusted for SUA, eGFR, FT3, and FT4. Participants were then stratified into 16 subgroups according to age (> 45 or ≤ 45), sex (male/female), smoking status, and the presence of hypertension, diabetes, dyslipidemia, obesity, or hyperuricemia to further explore the multifactorial subgroup associations between AIP and AVC. In addition, in the sensitivity analysis, participants with eGFR < 60 ml/min/1.73m^2^ were excluded, and the associations between AIP and AVC were reanalyzed in the three models above. AIP was also grouped according to its median, mean, and quartiles to jointly assess the stability of the correlation between AIP and AVC. However, to avoid type I error inflation due to multiple testing with various AIP grouping methods, we designated the quartile method as the primary analysis approach. Grouping analyses based on tertiles, medians, and means were considered sensitivity analyses and were not subjected to α-level correction (e.g., Bonferroni correction). The results of the sensitivity analyses were regarded as supportive evidence rather than independent confirmation of the primary hypothesis. Finally, the receiver operating characteristic (ROC) was used to evaluate the diagnostic value of AIP and traditional lipid indicators (TG, TC) for AVC, as well as the area under the curve (AUC) for each indicator. The DeLong test was applied to compare differences in AUCs between AIP and TG, TC, to assess whether AIP had significant incremental diagnostic value. Although the plasma atherogenic index (AIP) is mathematically derived from triglycerides (TG) and high-density lipoprotein cholesterol (HDL-C), multivariate logistic regression analysis showed that neither HDL-C nor low-density lipoprotein cholesterol (LDL-C) was significantly associated with the risk of aortic valve calcification (AVC) (both P > 0.05). However, to provide a complete biological baseline, we additionally included HDL-C and LDL-C in the ROC comparison. All AUC comparisons were performed using the DeLong test. All tests were two-sided, with P < 0.05 considered statistically significant.

## 3 Results

### 3.1 Clinical features according to AVC grouping

As shown in **Table 1**, the study included 1,752 participants. Based on AVC, participants were divided into two groups: the non-AVC group and the AVC group. Compared to the non-AVC group, participants in the AVC group had higher age, higher levels of monocyte count, RDW, FPG, HbA1c, TBIL, TG, eGFR, and AIP, while also having lower levels of lymphocyte count, platelet count, albumin, SUA, FT3, and FT4 (P < 0.05). No significant statistical differences were observed in other variables between the two groups (P > 0.05).

**Table 1:** Clinical characteristics based on AVC grouping.

Variables	Total population	Non-AVC	AVC	P value	Missing (%)
N	1752	1463	289		
Age, years	47.0 (30.0, 55.0)	46.0 (30.0, 55.0)	49.0 (31.0, 57.0)	0.032	0
Sex, n (%)				0.169	0
Male	1194 (68.2%)	1007 (68.8%)	187 (64.7%)		
Female	558 (31.8%)	456 (31.2%)	102 (35.3%)		
Smoking, n (%)				0.180	0
Yes	487 (27.8%)	416 (28.4%)	71 (24.6%)		
No	1265 (72.2%)	1047 (71.6%)	218 (75.4%)		
Hypertension, n (%)				0.255	0
Yes	580 (33.1%)	476 (32.5%)	104 (36.0%)		
No	1172 (66.9%)	987 (67.5%)	185 (64.0%)		
Diabetes, n (%)				0.657	0
Yes	353 (20.1%)	292 (20.0%)	61 (21.1%)		
No	1399 (79.9%)	1171 (80.0%)	228 (78.9%)		
Dyslipidemia, n (%)				0.114	0
Yes	614 (35%)	501 (34.2%)	113 (39.1%)		
No	1138 (65%)	962 (65.8%)	176 (60.9%)		
Obesity, n (%)				0.211	0
Yes	440 (25.1%)	359 (24.5%)	81 (28.0%)		
No	1312 (74.9%)	1104 (75.5%)	208 (72.0%)		
Hyperuricemia, n (%)				0.765	0
Yes	611 (34.9%)	508 (34.7%)	103 (35.6%)		
No	1141 (65.1%)	955 (65.3%)	186 (64.4%)		
BMI, kg/m^2^	25.5 (23.4, 28.0)	25.5 (23.4, 27.9)	25.9 (23.2, 28.3)	0.733	0
SBP, mmHg	124.0 (114.0, 137.0)	123.0 (114.0, 136.0)	126.0 (114.0, 138.5)	0.121	0
DBP, mmHg	77.0 (70.0, 85.0)	77.0 (70.0, 93.0)	78.0 (70.0, 86.0)	0.256	0
WBC, x10^9^/L	6.1 (5.2, 7.3)	6.1 (5.2, 7.3)	6.0 (5.0, 7.4)	0.569	0.23
Neutrophil, x10^9^/L	3.6 (2.8, 4.4)	3.6 (2.8, 4,4)	3.5 (2.7, 4,6)	0.828	0.23
Lymphocyte, x10^9^/L	1.9 (1.6, 2,3)	2.0 (1.6, 2,3)	1.8 (1.6, 2.3)	0.027	0.23
Monocytes, x10^9^/L	0.4 (0.3, 0.5)	0.4 (0.3, 0.5)	0.4 (0.4, 0.6)	0.047	0
Hemoglobin, g/L	153.0 (141.0, 161.0)	153.0 (141.0, 161.0)	152.0 (142.5, 160.0)	0.668	0
Hematocrit, %	46.1 (43.0, 48.4)	46.1 (42.9, 48.5)	46.0 (43.5, 48.2)	0.913	0.23
RDW, %	12.5 (12.1, 12.9)	12.4 (12.0, 12.9)	12.7 (12.3, 13.2)	<0.001	0.23
Platelet, x10^9^/L	236.0 (202.0, 272.0)	239.0 (105.0, 273.0)	220.0 (190.5, 259.0)	<0.001	0.23
ALT, U/L	21.8 (15.7, 32.1)	21.4 (15.4, 32.8)	22.5 (16.9, 29.6)	0.439	0
AST, U/L	20.7 (17.5, 25.0)	20.8 (17.4, 24.9)	20.4 (17.8, 25.3)	0.877	0
Albumin, g/L	45.2 (43.5, 47.0)	45.4 (43.6, 47.2)	44.5 (42.7, 46.0)	<0.001	0
TBIL, μmol/L	14.0 (10.8, 18.0)	13.9 (10.7, 18.0)	14.4 (11.2, 18.2)	0.178	0
Glucose, mmol/L	4.8 (4.6, 5.2)	4.8 (4.5, 5.2)	5.2 (4.8, 6.2)	<0.001	0
HbA1c, %	5.6 (5.3, 5.8)	5.6 (5.3, 5.8)	5.8 (5.6, 6.6)	<0.001	0
Total cholesterol, mmol/L	4.4 (3.9, 5.0)	4.4 (3.9, 5.0)	4.5 (4.0, 5.2)	0.042	0
Triglyceride, mmol/L	1.4 (0.9, 2.1)	1.3 (0.9, 2.0)	1.7 (1.2, 2.2)	<0.001	0
HDL-C, mmol/L	1.2 (1.0, 1.3)	1.2 (1.0, 1.3)	1.2 (1.0, 1.4)	0.726	0
LDL-C, mmol/L	2.9 (2.4, 3.4)	2.9 (2.5, 3.4)	2.9 (2.4, 3.5)	0.973	0
Scr, μmol/L	71.9 (61.0, 81.6)	72.5 (60.9, 82.0)	70.0 (63.0, 80.8)	0.898	0
Serum uric acid, μmol/L	369.4 (304.2, 435.1)	375.7 (309.0, 441.3)	343.0 (288.0, 403.9)	<0.001	0
eGFR, mL/min/1.73m²	103.3 (94.0, 114.4)	102.6 (92.9, 113.6)	107.6 (98.6, 117.6)	<0.001	0
FT3, pmol/L	5.3 (4.8, 5.8)	5.3 (4.9, 5.8)	5.1 (4.6, 5.5)	<0.001	0.51
FT4, pmol/L	17.2 (15.5, 18.8)	17.3 (15.5, 18.9)	16.8 (15.4, 18.4)	0.015	0.51
1-25(OH)D3, nmol/L	52.4 (44.9, 56.2)	52.4 (44.7, 56.8)	52.4 (45.2, 52.4)	0.234	28.3
Homocysteine, mg/L	13.4 (10.6, 13.4)	13.4 (10.6, 13.4)	13.4 (10.7, 13.4)	0.910	28.3
AIP	0.1 (-0.1, 0.3)	0.1 (-0.2, 0.3)	0.1 (0, 0.3)	<0.001	0

AVC, Aortic valve calcification; BMI, Body mass index; SBP, Systolic blood pressure; DBP, Diastolic blood pressure; WBC, White blood cell; RDW, Red cell distribution width; ALT, Alanine aminotransferase; AST, Aspartate aminotransferase; TBIL, Total bilirubin; HbA1c, Glycosylated hemoglobin; HDL-C, High-density lipoprotein cholesterol; LDL-C, Low-density lipoprotein cholesterol; Scr, Serum creatinine; eGFR, Estimated glomerular filtration rate; FT3, Free triiodothyronine; FT4, Free thyroxine; 1-25(OH)D3, 1,25-Dihydroxyvitamin D3; AIP, Atherogenic index of plasma.*Missing data: For variables with complete data (N = 1752), missing percentage is 0%. For WBC, Neutrophil, Hematocrit, RDW and Platelet (N = 1748), missing percentage is 0.23%. For FT3 and FT4 (N = 1743), missing percentage is 0.51%. For 1-25(OH)D3 and homocysteine (N = 1252), missing percentage is 28.3%. Missing values were imputed using mean imputation as the missing proportion was <30%.

### 3.2 Clinical characteristics grouped by the AIP tertiles

As shown in **Table 2**, participants were divided into three groups based on AIP tertiles: T1 (n = 579); T2 (n = 591); T3 (n = 582). Significant differences were observed among the three groups in terms of sex, smoking status, hypertension, diabetes, dyslipidemia, obesity, hyperuricemia, BMI, SBP, DBP, WBC, neutrophil count, lymphocyte count, monocyte count, hemoglobin, hematocrit, ALT, AST, albumin, TBIL, FPG, HbA1c, TG, TC, HDL-C, LDL-C, Scr, SUA, FT3, FT4, and AVC (all P < 0.05). Notably, the prevalence of AVC increased with higher AIP group levels (P < 0.001).

**Table 2:** Clinical features comparison based on AIP grouping.

Variables	T1	T2	T3	P value
N	579	591	582	
Age, years	46.0 (30.0, 55.0)	47.0 (30.0, 56.0)	47.0 (30.0, 55.0)	0.687
Sex, n (%)				<0.001
Male	316 (54.6%)	432 (73.1%)	446 (76.6%)	
Female	263 (45.4%)	159 (26.9%)	136 (23.4%)	
Smoking, n (%)				0.029
Yes	138 (23.8%)	180 (30.5%)	169 (29.0%)	
No	441 (76.2%)	411 (69.5%)	413 (71.0%)	
Hypertension, n (%)				<0.001
Yes	137 (23.7%)	197 (33.3%)	246 (42.3%)	
No	442 (76.3%)	394 (66.7%)	336 (57.7%)	
Diabetes, n (%)				0.016
Yes	94 (16.2%)	129 (21.8%)	130 (22.3%)	
No	485 (83.8%)	462 (78.2%)	452 (77.7%)	
Dyslipidemia, n (%)				<0.001
Yes	29 (5%)	127 (21.5%)	458 (78.7%)	
No	550 (95%)	464 (78.5%)	124 (21.3%)	
Obesity, n (%)				<0.001
Yes	32 (5.5%)	139 (23.5%)	269 (46.2%)	
No	547 (94.5%)	452 (76.5%)	313 (53.8%)	
Hyperuricemia, n (%)				<0.001
Yes	59 (10.2%)	206 (34.9%)	346 (59.5%)	
No	520 (89.8%)	385 (65.1%)	236 (40.5%)	
BMI, kg/m²	23.3 (21.3, 25.0)	26.0 (24.1, 27.8)	25.5 (23.8, 27.6)	<0.001
SBP, mmHg	120.0 (110.0, 133.0)	124.0 (115.0, 136.0)	127.0 (117.0, 140.0)	<0.001
DBP, mmHg	74.0 (70.0, 82.0)	78.0 (71.0, 86.0)	80.0 (72.0, 88.0)	<0.001
WBC, x10^9^/L	5.6 (4.7, 6.6)	6.2 (5.3, 7.2)	6.7 (5.7, 7.9)	<0.001
Neutrophil, x10^9^/L	3.1 (2.5, 4.0)	3.6 (2.9, 4,3)	3.9 (3.1, 4.7)	<0.001
Lymphocyte, x10^9^/L	1.8 (1.5, 2,1)	1.9 (1.6, 2,3)	2.1 (1.8, 2.5)	<0.001
Monocytes, x10^9^/L	0.39 (0.30, 0.47)	0.43 (0.35, 0.51)	0.47 (0.37, 0.56)	<0.001
Hemoglobin, g/L	143.0 (133.0, 155.0)	154.0 (144.0, 161.0)	158.0 (150.0, 164.0)	<0.001
Hematocrit, %	43.5 (40.7, 46.7)	46.2 (43.9, 48.4)	47.5 (45.2, 49.4)	<0.001
RDW, %	12.5 (12.1, 13.0)	12.4 (12.1, 12.9)	12.5 (12.1, 12.9)	0.606
Platelet, x10^9^/L	234.0 (201.0, 268.0)	234.0 (202.0, 269.0)	241.0 (203.0, 277.0)	0.232
ALT, U/L	16.4 (12.1, 22.5)	22.9 (16.8, 32.8)	27.0 (20.0, 39.7)	<0.001
AST, U/L	19.1 (16.4, 23.0)	20.9 (17.7, 25.1)	22.1 (19.1, 26.7)	<0.001
Albumin, g/L	44.8 (43.0, 46.7)	45.4 (43.5, 47.3)	45.4 (43.8, 47.0)	0.001
TBIL,μmol/L	13.9 (10.4, 17.9)	14.5 (11.4, 18.8)	13.8 (10.6, 17.5)	0.011
Glucose, mmol/L	4.7 (4.5,5.0)	4.8 (4.6, 5.2)	5.0 (4.7, 5.6)	<0.001
HbA1c, %	5.6 (5.3, 5.8)	5.6 (5.3, 5.8)	5.7 (5.4, 6.1)	<0.001
Total cholesterol, mmol/L	4.2 (3.7, 4.8)	4.4 (3.9, 5.0)	4.7 (4.1, 5.3)	<0.001
Triglyceride, mmol/L	0.8 (0.7, 1.0)	1.4 (1.2, 1.6)	2.54 (2.0, 3.5)	<0.001
HDL, mmol/L	1.3 (1.2, 1.5)	1.2 (1.0, 1.3)	1.1 (0.9, 1.2)	<0.001
LDL, mmol/L	2.6 (2.2, 3.1)	3.0 (2.5, 3.4)	3.1 (2.6, 3.5)	<0.001
Scr,μmol/L	64.2 (56.5, 77.6)	74.3 (64.0, 82.9)	74.6 (65.5, 83.6)	<0.001
Serum uric acid,μmol/L	320.8 (270.2, 385.4)	374.2 (314.1, 429.3)	411.8 (351.8, 474.8)	<0.001
eGFR, mL/min/1.73m²	111.6 (101.3, 120.1)	102.7 (95.0, 112.6)	97.4 (88.3, 106.3)	<0.001
FT3, pmol/L	5.1 (4.6, 5.6)	5.4 (5.0, 5.8)	5.4 (4.9, 6.0)	<0.001
FT4, pmol/L	17.2 (15.5, 18.8)	17.4 (15.7, 19.0)	17.0 (15.4, 18.5)	0.047
1-25(OH)D3, nmol/L	52.4 (47.5, 54.6)	52.4 (44.0, 55.6)	52.4 (43.1, 58.0)	0.590
Homocysteine, mg/L	13.4 (10.8, 13.4)	13.4 (10.5, 13.4)	13.4 (10.6, 14.0)	0.695
AVC, n%				<0.001
Yes	67 (11.6%)	102 (17.3%)	120 (20.6%)	
No	512 (88.4%)	489 (82.7%)	462 (79.4%)	

T1:AIP ≤ -0.06;T2:-0.06 < AIP ≤ 0.20;T3:AIP > 0.20. AVC, Aortic valve calcification; BMI, Body mass index; SBP, Systolic blood pressure; DBP, Diastolic blood pressure; WBC, White blood cell; RDW, Red cell distribution width; ALT, Alanine aminotransferase; AST, Aspartate aminotransferase; TBIL, Total bilirubin; HbA1c, Glycosylated hemoglobin; HDL-C, High-density lipoprotein cholesterol; LDL-C, Low-density lipoprotein cholesterol; Scr, Serum creatinine; eGFR, Estimated glomerular filtration rate; FT3, Free triiodothyronine; FT4, Free thyroxine; 1-25(OH)D3, 1,25-Dihydroxyvitamin D3; AIP, Atherogenic index of plasma.

### 3.3 Univariate logistic regression analysis of AVC

As shown in **Table 3**, univariate logistic regression analysis indicated that monocyte count, RDW, platelet count, albumin, TBIL, FPG, HbA1c, SUA, eGFR, FT3, FT4, and AIP were all associated with the risk of AVC (P < 0.05). Notably, for each one-unit increase in AIP, the risk of AVC increased by 98.5% (OR = 1.985, 95% CI 1.339–2.943, P = 0.001). Moreover, the risks of AVC in the T2 and T3 groups were 1.594 times (OR = 1.594, 95% CI 1.143–2.222, P = 0.006) and 1.985 times (OR = 1.985, 95% CI 1.435–2.745, P < 0.001) that of the T1 group, respectively. However, other variables showed no significant association with the risk of AVC (P > 0.05).

**Table 3:** Univariate logistic regression analysis of AVC.

Variables	OR	95% CI	P value
Age	1.008	1.000, 1.017	0.058
Male	0.830	0.637, 1.083	0.169
Smoking	0.820	0.613, 1.096	0.180
Hypertension	1.166	0.895, 1.518	0.255
Diabetes	1.073	0.787, 1.463	0.657
Dyslipidemia	1.233	0.951, 1.599	0.114
Obesity	1.198	0.902, 1.589	0.212
Hyperuricemia	1.041	0.800, 1.355	0.765
BMI	1.000	0.968, 1.033	0.983
SBP, mmHg	1.006	0.998, 1.013	0.125
DBP, mmHg	1.006	0.995, 1.017	0.294
WBC, x10^9^/L	1.035	0.962, 1.114	0.361
Neutrophil, x10^9^/L	1.059	0.965, 1.161	0.228
Lymphocyte, x10^9^/L	0.834	0.656, 1.060	0.137
Monocytes, x10^9^/L	3.942	1.723, 9.018	0.001
Hemoglobin, g/L	1.003	0.994, 1.011	0.500
Hematocrit, %	1.011	0.979, 1.043	0.510
RDW, %	1.234	1.085, 1.402	0.001
Platelet, x10^9^/L	0.995	0.992, 0.997	<0.001
ALT, U/L	0.996	0.989, 1.002	0.199
AST, U/L	0.998	0.987, 1.008	0.646
Albumin, g/L	0.880	0.841, 0.922	<0.001
TBIL, μmol/L	1.021	1.002, 1.040	0.029
Glucose, mmol/L	1.246	1.167, 1.331	<0.001
HbA1c, %	1.735	1.536, 1.960	<0.001
Total cholesterol, mmol/L	1.144	1.000, 1.309	0.050
Triglyceride, mmol/L	1.056	0.990, 1.126	0.096
HDL, mmol/L	1.223	0.766, 1.953	0.398
LDL, mmol/L	0.983	0.824, 1.174	0.852
Scr, μmol/L	1.006	0.998, 1.014	0.173
Serum uric acid, μmol/L	0.996	0.995, 0.998	<0.001
eGFR, mL/min/1.73m²	1.023	1.014, 1.032	<0.001
FT3, pmol/L	0.624	0.520, 0.748	<0.001
FT4, pmol/L	0.932	0.884, 0.984	0.010
1-25(OH)D3, nmol/L	0.997	0.989, 1.006	0.545
Homocysteine, mg/L	1.002	0.981, 1.023	0.876
AIP (Continuous variable)	1.985	1.339, 2.943	0.001
AIP (Three-quantile grouping)			
T1 (≤ -0.06)	Ref		
T2 (-0.06 < AIP ≤ 0.20)	1.594	1.143, 2.222	0.006
T3 (> 0.20)	1.985	1.435, 2.745	<0.001
P for trend			<0.001

AVC, Aortic valve calcification; BMI, Body mass index; SBP, Systolic blood pressure; DBP, Diastolic blood pressure; WBC, White blood cell; RDW, Red cell distribution width; ALT, Alanine aminotransferase; AST, Aspartate aminotransferase; TBIL, Total bilirubin; HbA1c, Glycosylated hemoglobin; HDL-C, High-density lipoprotein cholesterol; LDL-C, Low-density lipoprotein cholesterol; Scr, Serum creatinine; eGFR, Estimated glomerular filtration rate; FT3, Free triiodothyronine; FT4, Free thyroxine; 1-25(OH)D3, 1,25-Dihydroxyvitamin D3; AIP, Atherogenic index of plasma.

### 3.4 Multivariate logistic regression analysis of AIP and AVC

As shown in **Table 4**, in models 1 (adjusting only for age and sex), 2 (partially adjusted), and 3 (fully adjusted), for each 1-unit increase in AIP, the risk of AVC increased by 115.7% (OR = 2.157, 95% CI 1.445-3.218, P < 0.001), 59.0% (OR = 1.590, 95% CI 1.013-2.497, P = 0.044), and 146.0% (OR = 2.460, 95% CI 1.525-3.969, P < 0.001), respectively. In Model 1, the AVC risk in the AIP Q2, Q3, and Q4 groups was 1.623 times (OR = 1.623, 95% CI 1.080–2.439, P = 0.020), 2.405 times (OR = 2.405, 95% CI 1.622–3.567, P < 0.001), and 2.319 times (OR = 2.319, 95% CI 1.564–3.439, P < 0.001) that of the Q1 group, respectively. In Model 2, the AVC risk in the AIP Q2, Q3, and Q4 groups was 1.740 times (OR = 1.740, 95% CI 1.136–2.668, P = 0.011), 2.215 times (OR = 2.215, 95% CI 1.454–3.376, P < 0.001), and 2.063 times (OR = 2.063, 95% CI 1.337–3.182, P = 0.001) that of the Q1 group, respectively.

**Table 4:** Multivariate logistic regression analysis of AIP and AVC.

Variables	Model 1		Model 2		Model 3	
	OR (95% CI)	P value	OR (95% CI)	P value	OR (95% CI)	P value
AIP (Continuous variable)	2.157 (1.445, 3.218)	<0.001	1.590 (1.013, 2.497)	0.044	2.460 (1.525, 3.969)	<0.001
AIP (Quartiles grouping)						
Q1 (≤ -0.14)	Ref		Ref		Ref	
Q2 (-0.14< AIP ≤ 0.07)	1.623 (1.080, 2.439)	0.020	1.740 (1.136, 2.668)	0.011	2.103 (1.361, 3.248)	0.001
Q3 (0.07< AIP ≤0.27)	2.405 (1.622, 3.567)	<0.001	2.215 (1.454, 3.376)	<0.001	2.966 (1.918, 4.586)	<0.001
Q4 (> 0.27)	2.319 (1.564, 3.439)	<0.001	2.063 (1.337, 3.182)	0.001	3.229 (2.028, 5.143)	<0.001
P for trend		<0.001		0.002		<0.001

Model 1: adjusted for age and sex only; Model 2: adjusted for age, sex, monocytes, RDW, platelet, albumin, total bilirubin, glucose, and HbA1c; Model 3: adjusted for age, sex, monocytes, RDW, platelet, albumin, total bilirubin, glucose, HbA1c, uric acid, eGFR, free triiodothyronine, and free thyroxine. AVC, Aortic valve calcification; AIP, Atherogenic index of plasma; RDW, Red cell distribution width; HbA1c, Glycosylated hemoglobin; eGFR, Estimated glomerular filtration rate.

Furthermore, in the fully adjusted Model 3, the risk of AVC for the AIP Q2 group, Q3 group, and Q4 group was 2.103 times (OR = 2.103, 95% CI 1.361-3.248, P = 0.001), 2.966 times (OR = 2.966, 95% CI 1.918-4.586, P < 0.001), and 3.229 times (OR = 3.229, 95% CI 2.028-5.143, P < 0.001) that of the Q1 group, respectively.

### 3.5 Multivariate subgroup analysis

As shown in **Table 5**, subgroup analysis indicated that the correlation between AIP and AVC was significant in most subgroups. Specifically, in the subgroups of age ≤ 45 years and > 45 years, for each 1-unit increase in AIP, the risk of AVC increases by 172.9% (OR = 2.729, 95% CI 1.289-5.776, P = 0.009) and 142.9% (OR = 2.429, 95% CI 1.267-4.655, P = 0.007), respectively. At the same time, based on AIP tertile grouping, in the age ≤ 45 and > 45 subgroups, the AVC risk in the T3 group of AIP was 3.382 times and 2.707 times that of the T1 group, respectively (P < 0.05). In the male and female subgroups, for each 1-unit increase in AIP, the risk of AVC increased by 111.9% (OR = 2.119, 95% CI 1.145–3.924, P = 0.017) and 338.9% (OR = 4.389, 95% CI 1.925–10.003, P < 0.001), respectively, and the AVC risk in the T3 group of AIP was 3.060 times and 3.376 times that of the T1 group in the male and female subgroups, respectively (P < 0.05).

**Table 5:** Multivariate subgroup analysis of AIP and AVC.

Subgroups	AIP (T2 vs T1)		AIP (T3 vs T1)		AIP	
	OR (95% CI)	P value	OR (95% CI)	P value	OR (95% CI)	P value
Age						
≤ 45 years	2.203 (1.237, 3.923)	0.007	3.382 (1.812, 6.315)	<0.001	2.729 (1.289, 5.776)	0.009
> 45 years	2.450 (1.500, 4.003)	<0.001	2.707 (1.589, 4.612)	<0.001	2.429 (1.267, 4.655)	0.007
Sex						
Male	2.409 (1.474, 3.938)	<0.001	3.060 (1.816, 5.154)	<0.001	2.119 (1.145, 3.924)	0.017
Female	1.895 (1.044, 3.442)	<0.036	3.376 (1.745, 6.533)	<0.001	4.389 (1.925, 10.003)	<0.001
Smoking						
Yes	1.634 (0.780, 3.423)	0.193	1.609 (0.721, 3.590)	0.245	1.525 (0.563, 4.130)	0.406
No	2.423 (1.584, 3.707)	<0.001	3.530 (2.220, 5.614)	<0.001	2.745 (1.578, 4.777)	<0.001
Hypertension						
Yes	1.496 (0.768, 2.917)	0.237	2.413 (1.227, 4.748)	0.011	2.288 (1.045, 5.008)	0.038
No	2.686 (1.704, 4.236)	<0.001	3.077 (1.843, 5.140)	<0.001	2.596 (1.399, 4.817)	0.002
Diabetes						
Yes	1.906 (0.780, 4.657)	0.157	5.052 (1.971, 12.951)	0.001	5.170 (1.601, 16.699)	0.006
No	2.062 (1.377, 3.088)	<0.001	2.231 (1.433, 3.474)	<0.001	1.881 (1.108, 3.192)	0.019
Dyslipidemia						
Yes	1.316 (0.352, 4.922)	0.683	2.006 (0.570, 7.063)	0.278	0.990 (0.418, 2.344)	0.982
No	2.214 (1.470, 3.336)	<0.001	3.556 (1.974, 6.404)	<0.001	10.680 (4.115, 27.718)	<0.001
Obesity						
Yes	1.409 (0.300, 6.630)	0.664	2.846 (0.632, 12.812)	0.173	1.066 (0.397, 2.860)	0.899
No	2.353 (1.588, 3.487)	<0.001	2.436 (1.524, 3.893)	<0.001	3.123 (1.670, 5.841)	<0.001
Hyperuricemia						
Yes	1.781 (0.580, 5.464)	0.313	3.565 (1.193, 10.657)	0.023	1.636 (0.692, 3.865)	0.262
No	2.380 (1.581, 3.583)	<0.001	2.327 (1.396, 3.879)	0.001	3.139 (1.613, 6.109)	0.001

Subgroups analysis adjusted for age, sex, monocytes, RDW, platelet, albumin, total bilirubin, glucose, HbA1c, uric acid, eGFR, free triiodothyronine, and free thyroxine. AVC, Aortic valve calcification; AIP, Atherogenic index of plasma; RDW, Red cell distribution width; HbA1c, Glycosylated hemoglobin; eGFR, Estimated glomerular filtration rate.

In the non-smoking group, for each 1-unit increase in AIP, the risk of AVC increased by 174.5% (OR = 2.745, 95% CI 1.578–4.777, P < 0.001), and the risk of AVC in the T2 and T3 groups of AIP was 2.423 and 3.530 times that of the T1 group, respectively (P < 0.05). In the subgroups with and without hypertension, each 1-unit increase in AIP corresponded to a 128.8% (OR = 2.288, 95% CI 1.045–5.008, P = 0.038) and 159.6% (OR = 2.596, 95% CI 1.399–4.817, P = 0.002) increase in AVC risk, respectively, and in both subgroups, the T3 group of AIP had an AVC risk 2.413 and 3.077 times that of the T1 group, respectively (P < 0.05). In both the subgroups with and without diabetes, each 1-unit increase in AIP was associated with a 417.0% increase in the risk of AVC (OR = 5.170, 95% CI 1.601–16.699, P = 0.006) and an 88.1% increase (OR = 1.881, 95% CI 1.108–3.192, P = 0.019), respectively. Moreover, in both subgroups, the T3 group of AIP had an AVC risk 5.052 times and 2.231 times higher than that of the T1 group (P < 0.05). In the subgroups without dyslipidemia, obesity, and hyperuricemia, each 1-unit increase in AIP also significantly elevated the risk of AVC, with the T3 group’s AVC risk being 3.556 times, 2.436 times, and 2.327 times that of the T1 group, respectively (P < 0.05). In addition, in the hyperuricemia subgroup, the T3 group of AIP had an AVC risk 3.565 times higher than that of the T1 group (P < 0.05).

### 3.6 Multivariable logistic regression analysis of AIP and AVC: excluding eGFR 60 mL/min/1.73m²

As shown in **Table 6**, after excluding individuals with eGFR < 60 ml/min/1.73m^2^, the risk of AVC was significantly associated with AIP in all three models. In the fully adjusted model, for every 1-unit increase in AIP, the risk of AVC increased by 143.2% (OR = 2.432, 95% CI 1.507-3.926, P < 0.001). At the same time, based on the AIP quartile grouping, the AVC risks for the Q2, Q3, and Q4 groups are 2.086, 2.928, and 3.172 times that of the Q1 group, respectively (P < 0.05).

**Table 6:** Sensitivity analysis: excluding eGFR < 60 ml/min/1.73m^2.^.

Variables	Model 1		Model 2		Model 3	
	OR (95% CI)	P value	OR (95% CI)	P value	OR (95% CI)	P value
AIP (Continuous variable)	2.176 (1.448, 3.246)	<0.001	1. 604 (1.022, 2.518)	0.040	2.432 (1.507, 3.926)	<0.001
AIP (Quartiles grouping)						
Q1 (≤ -0.14)	Ref		Ref		Ref	
Q2 (-0.14< AIP ≤ 0.07)	1.653 (1.099, 2.485)	0.016	1.811(1.184, 2.771)	0.006	2.086 (1.351, 3.211)	=0.001
Q3 (0.07< AIP ≤0.27)	2.426 (1.635, 3.599)	<0.001	2.345 (1.544, 3.562)	<0.001	2.928 (1.895, 4.525)	<0.001
Q4 (> 0.27)	2.351(1.585, 3.486)	<0.001	2.253(1.472, 3.447)	<0.001	3.172(1.933, 5.048)	<0.001
P for trend		<0.001		<0.001		<0.001

Model 1: adjusted for age and sex only; Model 2: adjusted for age, sex, monocytes, RDW, platelet, albumin, total bilirubin, glucose, and HbA1c; Model 3: adjusted for age, sex, monocytes, RDW, platelet, albumin, total bilirubin, glucose, HbA1c, uric acid, eGFR, free triiodothyronine, and free thyroxine. AVC, Aortic valve calcification; AIP, Atherogenic index of plasma; RDW, Red cell distribution width; HbA1c, Glycosylated hemoglobin; eGFR, Estimated glomerular filtration rate.

### 3.7 Sensitivity analysis: based on different AIP threshold values

The sensitivity analysis was shown in **Table 7**, participants were divided into Low AIP group (≤ 0.07) and High AIP group (> 0.07) based on the median AIP. In this categorization, the risk of AVC in the High AIP group was 1.805 times, 1.560 times, and 1.996 times higher than in the Low AIP group in model 1, model 2, and model 3, respectively (P < 0.05). Furthermore, based on the average AIP value, participants were divided into Low AIP group (≤ 0.08) and High AIP group (> 0.08). The risk of AVC in the High AIP group was found to be 1.836 times, 1.611 times, and 2.067 times higher in model 1, 2, and 3, respectively, compared to the Low AIP group (P < 0.05). Finally, when AIP was divided into tertiles: T1 (AIP ≤ -0.06), T2 (-0.06 < AIP ≤ 0.20), T3 (AIP > 0.20), after adjusting for all confounding factors, the AVC risk in T2 and T3 was 2.151 times and 2.807 times higher than in T1, respectively (P < 0.05).

**Table 7:** Sensitivity analysis: grouping based on different cutoff values of AIP.

Variables	Model 1		Model 2		Model 3	
	OR (95% CI)	P value	OR (95% CI)	P value	OR (95% CI)	P value
AIP (Median grouping)						
Low AIP (≤ 0.07)	Ref		Ref		Ref	
High AIP (> 0.07)	1.805 (1.389, 2.346)	<0.001	1.560 (1.171, 2.080)	0.002	1.996 (1.471, 2.709)	<0.001
AIP (Mean grouping)						
Low AIP (≤ 0.08)	Ref		Ref		Ref	
High AIP (> 0.08)	1.836 (1.414, 2.383)	<0.001	1.611 (1.220, 2.127)	0.001	2.067 (1.522, 2.805)	<0.001
AIP (Tertiles grouping)						
T1 (≤ -0.06)	Ref		Ref		Ref	
T2 (-0.06 < AIP ≤ 0.20)	1.697 (1.211, 2.378)	0.002	1.691 (1.185, 2.413)	0.004	2.151 (1.489, 3.108)	<0.001
T3 (> 0.20)	2.140 (1.536, 2.983)	<0.001	1.863 (1.289, 2.691)	0.001	2.807 (1.882, 4.187)	<0.001
P for trend		<0.001		0.002		<0.001

Model 1: adjusted for age and sex only; Model 2: adjusted for age, sex, monocytes, RDW, platelet, albumin, total bilirubin, glucose, and HbA1c; Model 3: adjusted for age, sex, monocytes, RDW, platelet, albumin, total bilirubin, glucose, HbA1c, uric acid, eGFR, free triiodothyronine, and free thyroxine. AVC, Aortic valve calcification; AIP, Atherogenic index of plasma; RDW, Red cell distribution width; HbA1c, Glycosylated hemoglobin; eGFR, Estimated glomerular filtration rate.

### 3.8 ROC analysis of AIP, TG, TC, HDL-C and LDL-C for identifying AVC

**Figure 1:**
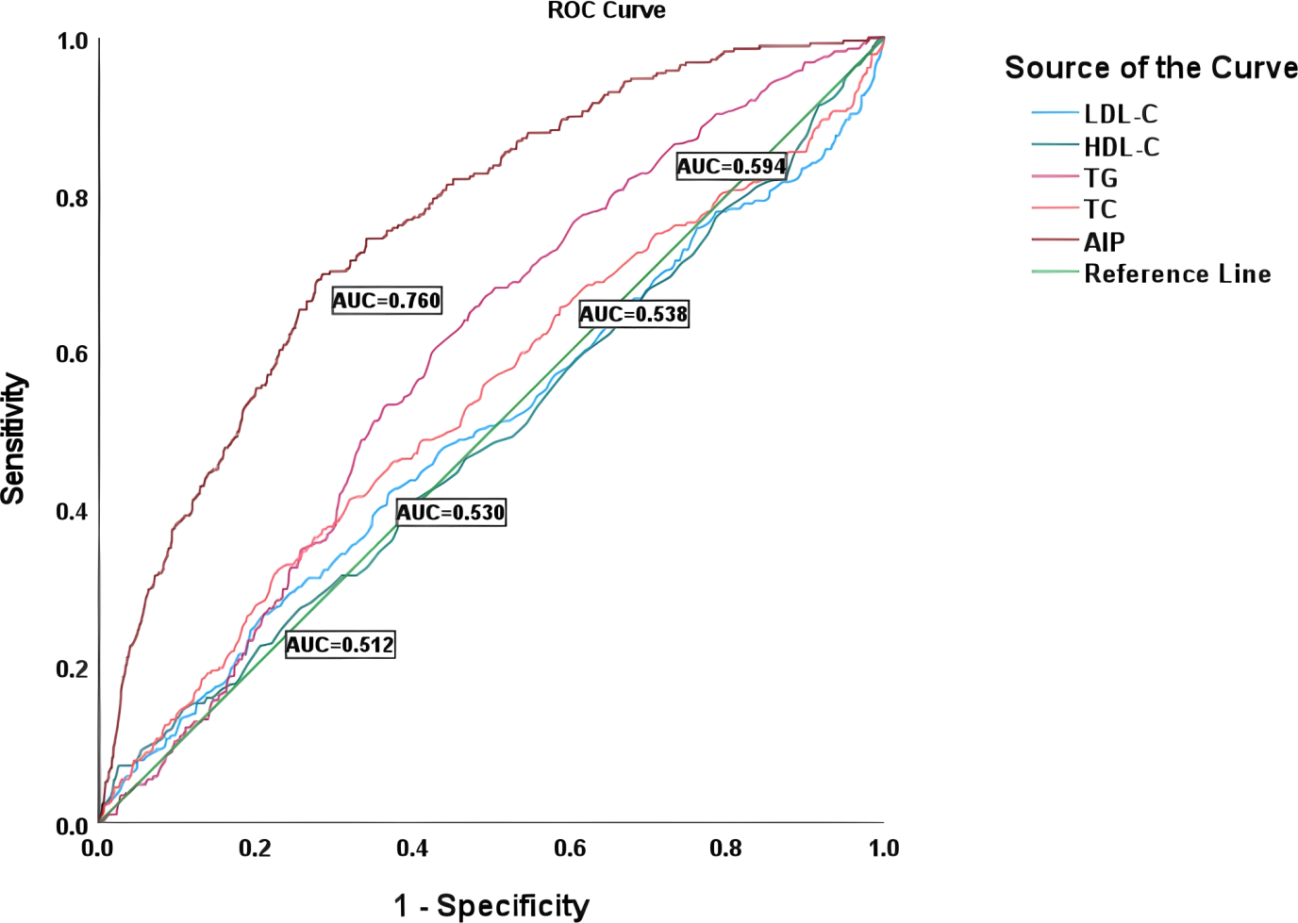
ROC curves of AIP, TG, TC, HDL-C and LDL-C for AVC. AIP, Atherogenic index of plasma; AVC, Aortic valve calcification; AUC, Area under the curve; TG, Triglycerides; TC, Total cholesterol; HDL-C, High-density lipoprotein cholesterol; LDL-C, Low-density lipoprotein cholesterol.

ROC curve analysis showed that the AUC of AIP for identifying AVC was 0.760 (95% CI 0.732–0.789, P < 0.001). The AUCs of traditional single lipid indicators were as follows: TG, 0.594 (95% CI 0.558–0.630); TC, 0.538 (95% CI 0.501–0.575); HDL-C, 0.512 (95% CI 0.474–0.550), and LDL-C, 0.530 (95% CI 0.492–0.568). According to the DeLong test, the AUC of AIP was significantly higher than those of TG,TC,HDL-C and LDL-C (P < 0.05)., indicating that AIP had a markedly superior discriminatory ability for AVC compared to traditional single lipid indicators (**Figure 1**).

## 4 Discussion

In this real-world study based on hospital data, we employed various statistical methods to evaluate the correlation between AIP and AVC risk in the general population. The results showed that even after adjusting for multiple confounding factors, AIP remained significantly associated with AVC risk, and this association was consistently observed in the vast majority of subgroup and sensitivity analyzes. Furthermore, when grouping based on multiple AIP cutoff values, the high-AIP group consistently exhibited a significantly increased risk of AVC. Finally, ROC curve analysis revealed that AIP had a moderate diagnostic value for AVC. These findings indicate that AIP holds important clinical value for the risk assessment and management of AVC in the general population, suggesting that more attention should be paid to AIP when assessing AVC risk in this population. However, as this is a single-center, retrospective observational cohort study, further research is needed to explore the potential association between the two.

The value of AIP as a biomarker for cardiovascular disease risk is increasingly gaining attention. A cross-sectional study based on the National Health and Nutrition Examination Survey (NHANES) database found a significant association between AIP and AAC. After adjusting for confounders, each 1-unit increase in AIP was associated with approximately a two-fold increased risk of AAC, and this association remained stable across subgroups with different age, gender, hypertension, diabetes, and other factors (14). In contrast, our study also found that after adjusting for confounders, the risk of AVC increased with higher AIP, and the results remained consistent across different subgroups. This suggests that the lipid metabolism disorder reflected by AIP may be a common risk factor for systemic vascular calcification. Furthermore, several studies have confirmed the association between AIP and CAC as well as carotid atherosclerosis. Li et al. found in a study involving 32,992 healthy screening participants that AIP levels were significantly higher in patients with CAC and carotid atherosclerosis, and AIP had a particularly strong predictive value for the co-occurrence of both lesions (AUC reached 0.91) (13). The study also revealed a dose-response relationship between AIP and atherosclerotic burden, with the highest quartile of AIP having more than twice the risk of CAC compared to the lowest quartile. Another study further showed that AIP is not only associated with CAC but also partially mediates the relationship between CAC and MACEs, with a mediation proportion of 8.16%-16.5%. This finding suggests that AIP may not merely be a risk marker for CAC but could also play a key pathological role linking subclinical atherosclerosis to clinical cardiovascular events (31).

Based on the above, this study extends the research perspective from the coronary arteries and aorta to the aortic valve, analyzing the impact of AIP on valve calcification. This aligns with a recent study on elderly patients with degenerative heart valve disease, which indicated that AIP is an independent predictor of valve calcification, further reinforcing the clinical value of AIP in assessing the risk of valve calcification (23). Additionally, a recent meta-analysis by Assempoor et al., which reviewed 51 observational studies involving over 200,000 subjects, showed that individuals with high AIP levels have a 179% increased risk of coronary artery disease, a 128% higher risk of developing CAC, and a significant association with multivessel disease and plaque progression risk (32). These findings further solidify the clinical value of AIP, and in line with the conclusions of this study, suggest that AIP may serve as a key biological marker linking lipid metabolism disorders to cardiovascular endpoint events.

Pathologically, AVC differs from arterial calcification, primarily affecting the fibrous layer of the valve leaflets rather than the intimal layer involved in atherosclerosis. Mechanistically, AIP, as the logarithmic transformation of the TG to HDL-C ratio, directly reflects the accumulation of TG-rich lipoproteins and HDL dysfunction. TG-rich lipoproteins and their remnants can penetrate the endothelial barrier, undergo oxidative modification, be engulfed by macrophages to form foam cells, release pro-inflammatory cytokines, and activate valvular interstitial cells to undergo osteogenic-like transformation (33, 34). When AIP is elevated, HDL is not only reduced in quantity but, more importantly, its antioxidant, anti-inflammatory, and cholesterol efflux functions are significantly impaired (35). Notably, apolipoprotein A-I (apoA-I), the core component of HDL, can undergo conformational changes under pathological conditions such as oxidative stress or shear stress, assembling into amyloid protofibrils. The exposed acidic amino acid residues on their surface can bind Ca²^+^ and promote the formation of calcium phosphate microcrystals, directly driving ectopic deposition of hydroxyapatite in valvular interstitial tissue. This process may be more closely associated with valvular calcification than with traditional arterial plaque formation (36). In addition, elevated AIP is often accompanied by insulin resistance, which can directly drive valvular interstitial cells toward osteogenic differentiation by activating transcription factors such as runt-related transcription factor 2 and bone morphogenetic protein 2 (37, 38). Meanwhile, AIP is significantly associated with various inflammatory markers (such as monocyte count and RDW), suggesting that it may continuously promote the calcification process by maintaining a low-grade inflammatory state (39–41). This aligns with the clinical features of patients in the AVC group in this study, namely increased monocyte count and RDW, along with decreased lymphocyte count and albumin.

Based on these mechanistic insights, our study further demonstrates that AIP, as an easily accessible biomarker, provides additional clinical value in risk stratification. AIP is derived from routine blood tests, requiring no extra cost or radiation exposure, and can aid in identifying high-risk populations for AVC in primary care settings, where echocardiography or cardiac CT is not readily available. Notably, our ROC analysis shows that AIP’s ability to detect AVC is significantly superior to traditional single lipid indicators (TG and TC). In univariate analysis, the associations of TG and TC with AVC were only marginal, whereas AIP, as a composite indicator, showed stronger correlations. Moreover, although HDL-C and LDL-C were not significantly associated with AVC in multivariate regression (both P > 0.05), we still included them in the ROC comparison. The results indicate that AIP’s AUC is significantly higher than both. These findings confirm that AIP can capture comprehensive information on lipid metabolic abnormalities that cannot be reflected by a single lipid marker, supporting its role not only as a statistically significant correlated indicator but also as a biomarker that offers additional clinical value in AVC risk assessment compared with traditional single lipid parameters.

It is worth noting that the subgroup analysis of this study shows that the association between AIP and AVC is significantly stronger in females than in males, a finding that aligns with recent trends reported in the literature. An analysis by Su et al. based on the NHANES database, the association between AIP and AAC was found to be statistically significant only in females. In females, for every 1-unit increase in AIP, the risk of severe AAC increased by 9.37 times, whereas no significant association was observed in males (42). This gender difference may be due to the protective effect of estrogen on the cardiovascular system before menopause. After menopause, the rapid decline in estrogen levels leads to adverse changes in lipid metabolism, making the increase in AIP more pronounced (38). HDL-C in females may be influenced by hormone levels. Research has shown that the protective effect of HDL-C on arterial calcification is modified by estradiol levels (43). However, in females with lower estrogen levels, this protective effect disappears and may even show an opposite trend. In addition, compared to men, the pathophysiological process of valve calcification in women may have certain particularities, with a higher sensitivity to lipid metabolism disorders (44). The aforementioned findings have implications for clinical practice: when assessing the risk of AVC in women, AIP should be considered a more focused indicator.

Although this study confirmed the association between AIP and AVC in the general population, some limitations are inevitably present. First, this study was a single-center, retrospective, retrospective observational cohort study, the results may lack sufficient statistical power, and the conclusions may not be easily generalized to other populations. Second, although the sample size of this study was relatively large, the incidence of AVC events was low, and in subgroup analyzes, the sample size of each subgroup was further reduced, which may lead to some type I errors. Third, although this study controlled for many confounding factors, some potential risk factors may still not have been adjusted for, such as clinical susceptibility, dietary factors, environmental factors, and occupational health factors. Fourth, in this study, AIP was calculated only from individually measured TG and HDL-C, without dynamic monitoring; therefore, the effect of dynamic changes in AIP and prolonged exposure levels on AVC cannot be determined. Fifth, although AVC was considered the primary outcome in this study, it was assessed by echocardiography and not comprehensively evaluated using cardiac CT scans or contrast-enhanced imaging. Sixth, multiple AIP grouping methods (quartiles as primary; tertiles, median, mean as sensitivity) were used to test the same hypothesis. Although the primary analysis was pre-specified, sensitivity analyses were not adjusted for multiple testing, which may increase the risk of Type I errors. Thus, sensitivity results should be interpreted cautiously. The main conclusions are based on quartile grouping, and the overall consistency across different methods supports the robustness of the findings. Seventh, this study did not include lipid-lowering drug use (e.g., statins, fibrates) as a covariate. Since AIP is derived from TG and HDL-C, and these drugs can alter both lipid parameters and AVC progression, the lack of adjustment may introduce confounding. We acknowledge this as a major limitation. If drug users are concentrated in the high AIP group and the drugs reduce both AIP and AVC risk, our OR estimates may be biased toward zero, potentially underestimating the true association. Due to incomplete medication records and inability to confirm adherence in this retrospective design, effective adjustment was not possible. Readers should interpret the results with caution, and future prospective studies should account for lipid-lowering therapy to clarify its impact on the AIP-AVC association. Eighth, an unexpected finding is that the AVC group had significantly higher eGFR than the non-AVC group, and univariate analysis showed eGFR as a positive correlate of AVC risk (OR = 1.023, P < 0.001), contradicting the conventional view that reduced eGFR promotes calcification. After repeated data verification, we confirmed that this result accurately reflects the current sample. Potential explanations include regional/population-specific characteristics (Xinjiang, multi-ethnic region), limited sample size (especially in AVC group), and preserved renal function of participants (eGFR ≥ 30 mL/min/1.73m²). Unmeasured confounders (e.g., medications, diet, parathyroid hormone) or chance might also contribute.Ninth, this study did not include coronary heart disease and heart failure in the multifactorial analysis. Although these two conditions are known to promote aortic valve calcification, re-analysis showed that there were only about 80 cases of coronary heart disease in the study population, with only a few cases having concomitant heart failure, leading to a highly uneven distribution between groups and resulting in unstable statistical outcomes. Therefore, we did not include these two variables in the final model. This decision may limit the generalizability of the study results, and the conclusions should be interpreted with caution. In the future, it will be necessary to expand the sample size to include more such patients in order to further clarify the independent association between AIP and AVC. Future large-scale, multicenter prospective studies are needed to clarify the true eGFR-AVC relationship.In summary, these limitations have to some extent restricted the further promotion and application of this study. In the future, we will improve the research design, address the shortcomings mentioned above, and further explore the relationship between the two.

## 5 Conclusion

This study confirms that AIP is significantly and independently positively correlated with AVC risk in the general population, and this association remains stable across different subgroups and sensitivity analyzes. Furthermore, AIP has a moderate diagnostic value for AVC. These findings suggest that AIP could serve as a novel associative biomarker for AVC risk assessment, providing new insights for early screening and intervention of AVC. Future large-scale prospective studies are needed to further validate the conclusions of this study and explore the impact of AIP-guided intervention strategies on AVC clinical outcomes.

## Data Availability

The raw data supporting the conclusions of this article will be made available by the authors, without undue reservation.
